# Social prescribing: an inadequate response to the degradation of social care in mental health

**DOI:** 10.1192/bjb.2023.61

**Published:** 2024-02

**Authors:** Rob Poole, Peter Huxley

**Affiliations:** Bangor University, Bangor, UK

**Keywords:** Social deprivation, social functioning, social prescribing, psychosocial interventions, social care

## Abstract

Social prescribing is poorly defined and there is little evidence for its effectiveness. It cannot address the social determinants of mental health and it is unlikely to produce enduring change for that part of the population that suffers the worst physical and mental health, namely the most deprived and marginalised. It has emerged at a time of growing health inequity. This has occurred alongside the neglect of social care and of the social aspects of mental health intervention. Social prescribing gives a false impression of addressing social factors, and as such is counterproductive. We can do better than this.

As co-directors of a research centre focused on the social aspects of mental health, we are troubled that social prescribing is being hyped in a way that is entirely inappropriate. In this paper, we suggest that current enthusiasm for social prescribing is wildly disproportionate to the very weak evidence for its effectiveness. Furthermore, overemphasis on social prescribing is seriously unhelpful in addressing the social origins of mental ill health. Although Marmot^1^ separates these into immediate causes and upstream ‘causes of the causes’, we believe that it is more fruitful to think in terms of constellations of disadvantage, where structural, environmental and experiential adversity interact in ways that are toxic to mental health. It is our contention that social prescribing has developed as a non-medical intervention after decades of underfunding and restrictive redefinition of social care in mental health. It is no solution to the serious problems that this has caused.

## What is social prescribing and what is its purpose?

Since the financial crisis of 2008, the physical and mental health of a significant proportion of the UK population has worsened to the point where life expectancy has reduced for some people.^2^ Political choices have increased inequality, creating downward pressure on low incomes, increasing the taxation burden for the poorest part of the population, systematically destroying manufacturing industries and their associated communities, defunding social infrastructure and generally degrading the social fabric for that part of the population that has always experienced the greatest burden of mental and physical ill health.

Social prescribing has emerged without any clear theoretical underpinning, and it is hard to say what exactly it is or what outcomes it achieves. The literature is short on hard outcome measures. Reviewing the evidence on social prescribing,^3,4^ it is striking that the term has no generally agreed definition. Like the term ‘assertive outreach’ before it, it refers to a heterogeneous group of interventions. Most of these interventions involve some form of link worker (there is a plethora of terms for the role) who brokers an individualised package of non-medical activity for the patient. The intervention is typically time-limited. We suggest that ‘facilitated social activity of limited duration prescribed by a medical practitioner’ is a reasonable working definition.

Faced with increasing numbers of patients whose ill health has a clear connection to their social environment, the concept of social prescribing has obvious attractions for practitioners who are uncomfortable with medicating the effects of adversity. Social prescribing is celebrated and promoted by bodies as diverse as NHS England, MIND and the Royal College of Psychiatrists.^5^ Journal articles, position papers and policies that promote social prescribing invariably support it through statements like ‘socioeconomic factors have consistently been found to have a greater impact on health than healthcare’.^6^ Such documents also refer to ‘emerging evidence’ that social prescribing is effective. Unfortunately, the juxtaposition of the two main statements creates a non sequitur. The fact that social factors cause ill health does not mean that any social intervention, no matter how limited in scope, is bound to be effective, and ‘emerging evidence’ is a euphemism for ‘inadequate evidence that favours our viewpoint’.

A recent review of the social prescribing literature used discourse analysis to examine the claims that are made for it.^7^ This suggested that there are three main discourses concerning social prescribing: that it is an appropriate response to health problems with social determinants; that it enhances self-management and reduces reliance on healthcare; and that it is a way of restoring person-centredness into primary care. It seems to us that, of these, only the self-management discourse is plausible. Even this carries the proviso that it is only likely to help people with straightforward problems who have sufficient personal resources to maintain any activity after the prescription expires. As a response to social adversity, especially in the context of severe mental illness, it is completely, indeed farcically, inadequate. Far from making primary care more patient-centred, it appears to us to further constrain the role of general practitioners to signposting. In failing to acknowledge the complexity of adverse social conditions, it weakens rather than strengthens the contextualisation of health problems. It is not that we are against, for example, free gym membership for people with metabolic syndrome. However, it will make little difference if it is not sustained and it will not help them to overcome their housing problems.

## The separation of ‘health’, ‘social’ and ‘care’

The role of social factors in causing and influencing illness has been subject to empirical study since the work of Querido^8^ in the 1950s. The biopsychosocial model was first proposed by Engel in 1977.^9^ Engel was a psychosomatic physician, and he intended his new model to apply to the whole of medicine. It has been psychiatry that has adopted it most enthusiastically; the Royal College of Psychiatrists endorses it as the primary framework for clinical practice. In the 21st century, Sir Michael Marmot is celebrated as one the most eminent medical authorities in the world, having spent his career producing highly influential epidemiological research on health inequity and inequality.^1^ It might be supposed from this history that an awareness of social aspects of illness and its treatment is by now firmly embedded in clinical practice, especially with regard to mental health. Instead, it is sad to report that social intervention has been systematically neglected for decades. In our opinion, British healthcare policymakers, including the medical Royal Colleges, have colluded with a distortion of the concept of ‘social’ to the point where current orthodoxy has a de facto role in the marginalisation within care systems of those groups of people who are most likely to suffer chronic ill health. Neither social care in general, nor social prescribing in particular, are intrinsically bad things, but, as currently conceptualised, they are completely inadequate responses to social need. They pay lip service to the importance of social intervention in a way that keeps important concepts well away from biomedicine, isolating interventions that are known to be effective from proper funding.

The past 40 years have been marked by repeated major reorganisations of health and social care services in the UK. These have been associated with a progressive shift away from state provision of long-term care, which has been as relabelled ‘social care’. The National Health Service and Community Care Act 1990 led to changes whereby local authorities became the brokers and care managers of social care, but not necessarily the direct providers, which facilitated progressive reductions in funding for long-term care and the narrowing of the scope of state-funded social intervention. Long-term care is now mainly provided by huge but precariously funded private health and social care sectors.

## The degrading of social care

As a result of these, and other, changes, ‘social care’ is mostly not social at all. Instead, in the mental health context, the term largely refers to either very limited provision at arms length in the voluntary sector or institutional placement for people with chronic ill health. The latter is justified by an implicit assumption that, in the absence of definitive technological intervention, positive change is impossible. The great hopeful movement that deinstitutionalised residents of large mental hospitals has been betrayed, and people who once were neglected in hospitals are now neglected in a virtual asylum of privatised health or social care.^10^

Under this new, undeclared, model, ‘healthcare’ has become constrained, confined to the provision of NICE approved interventions with known outcomes for specific diagnoses. The complex needs of people in social care mostly arise from physical and mental illness or disability, so that ‘social care’ means low-cost provision for people with chronic problems. The sequestration and neglect of social aspects of care and treatment have provoked a reaction, often articulated in calls for ‘holistic care’. This is distinguished from an undefined medical model, which is blamed for limitations to modern healthcare that actually have their origins in marketisation.

So it has come about that ‘social care’ is a euphemism for the withdrawal of resource from people with the greatest health needs. Previously, hospitals were places that offered care to people, staffed by qualified nurses with a commitment to bodily and psychological support rather than a primary role in deploying technologies. A change occurred with UK's embrace of neo-liberalism in the 1980s, which was associated with the monetisation of health intervention and removal of the state (and the collectivist mindset) from the care of chronically ill or disabled people. Nursing became a graduate profession with an increasingly technological role, and bodily and psychological support was delegated to unqualified staff. This has been associated with recurrent scandals over the quality of care. Having separated care and intervention, the former has been labelled ‘social’, making it easier to defund, focusing ‘health’ on atomised packages of intervention. This works well for the wealthy who get one disease at a time and very badly for poorer people who develop multiple health problems. Among all of this relabelling, the central role of social intervention in improving broadly defined health has been all but forgotten.

We worked in large mental hospitals, and we have no nostalgia for them. We belong to a generation of mental health professionals that had a strong commitment to caring for people in, or close to, their communities, taking their psychological and social contexts as seriously as biological aspects of their ill health. This demanded a robust understanding of an integrated biopsychosocial model. A care process that has sequestrated the social from the medical and the psychological is entirely inimical to this type of practice. Dispiritingly, the current model has led to a situation where psychiatrists work in isolation in clinics, tinkering with medications rather than playing a meaningful part in forming and delivering strategic clinical plans that have been co-produced with patients and their families. Social prescribing is one more decontextualised tool to add to the modern psychiatrist's miserably limited toolkit.

## Social prescribing is not a solution

In their discourse analysis, Calderón-Larrañaga et al^7^ suggest that in the literature, social prescribing is inappropriately framed as a ‘solution’ to complex problems and that this obscures any useful roles it might have. We agree with this. We do not suggest that social prescribing is harmful for the individuals who experience it. However, we do believe that it is extraordinary to make an ill-defined intervention a major component in mental health service planning, especially in the absence of evidence on outcomes or even clarity over its purpose. There are real potential harms beyond individuals.

First, it isolates the medical from the social aspects of care. It encourages practitioners to regard social factors as someone else's business, further fragmenting care provision.

Second, it gives a false impression of doing something about the social determinants of health. At an individual level, it is known that many social interventions are effective for the duration of staff involvement. Gains are lost thereafter. Social timescales are lengthy. We strongly support evidenced social interventions, but they must continue long enough to have a persistent effect.

Third, many structural and infrastructural interventions can make a difference to the health of communities, but they usually benefit whole populations, rather than just those who have an established mental health problem. In contrast, social interventions that can be delivered at low cost, safely away from health budgets, are bound to find favour with policymakers, even if they cannot be shown to make any real difference to health in the long run.

Fourth, as a policy label, the adjective ‘social’ has come to mean ‘not the responsibility of the state/health service’. If people do not take the opportunities that social prescribing offers them, well, that's their choice. There is no such thing as society, it's all down to individuals and their families. In reality, the obstructions to deprived people making good and enduring lifestyle choices are huge. You are unlikely to go jogging if the streets of your neighbourhood are unsafe. Temporary free access to facilities will not have much impact if you lack the resources to pay for them when public transport costs and the childcare costs are included, or if the charges become your responsibility when the prescription expires.

It is our professional responsibility to show a level of commitment to social prescribing that is proportional to the strength of the evidence for its effectiveness. The real problem here is that UK governments have been unwilling to commit resources to health and social care at the same level as other high-income countries. The primary determinants of who gets ill and who stays well (whether physically or mentally) are social, and among these, poverty is by far the most important. This means that the state is the only plausible source of adequate funding of care for the majority of people who need it. Social intervention is potent and important but it requires enduring funding. Against this background, social prescribing is, at best, a sop. At worst, it is the latest in a long series of efforts to transform the social into matters of individual responsibility.

## Conclusions

In accordance with Tudor Hart's inverse care law,^11^ the people who most benefit from social prescribing are likely to be those with the least severe and complex health needs. We can do better than this, but it will require us to jump off the social prescribing bandwagon. We should learn the lesson of assertive outreach. Disillusionment with that deliberately ill-defined model was followed by a loss of interest in supporting people with chronic mental illness in the community.^12^ It is now possible to see that the failure of assertive outreach ultimately had a role in reinstitutionalisation in the private sector. In place of uncritical advocacy of social prescribing, we need a bold movement that promotes evidence-based social intervention to improve the mental health of that section of the population that is most likely to become unwell.

## Data Availability

Data availability is not applicable to this article as no new data were created or analysed in this study.

## About the authors

**Rob Poole**, FRCPsych, is Professor of Social Psychiatry in the Centre for Mental Health and Society, Bangor University, Bangor, UK. **Peter Huxley**, PhD, is Professor of Mental Health Research in the Centre for Mental Health and Society, Bangor University, Bangor, UK.
